# Comparative proteomic profiling of refractory/relapsed multiple myeloma reveals biomarkers involved in resistance to bortezomib-based therapy

**DOI:** 10.18632/oncotarget.11059

**Published:** 2016-08-04

**Authors:** Dominik Dytfeld, Magdalena Luczak, Tomasz Wrobel, Lidia Usnarska-Zubkiewicz, Katarzyna Brzezniakiewicz, Krzysztof Jamroziak, Krzysztof Giannopoulos, Anna Przybylowicz-Chalecka, Blazej Ratajczak, Joanna Czerwinska-Rybak, Adam Nowicki, Monika Joks, Elzbieta Czechowska, Magdalena Zawartko, Tomasz Szczepaniak, Norbert Grzasko, Marta Morawska, Maciej Bochenek, Tadeusz Kubicki, Michalina Morawska, Katarzyna Tusznio, Andrzej Jakubowiak, Mieczysław Komarnicki

**Affiliations:** ^1^ Department of Hematology and Bone Marrow Transplantation, Poznan University of Medical Sciences, Poznan, Poland; ^2^ nstitute of Bioorganic Chemistry, Polish Academy of Sciences, Poznan, Poland; ^3^ Department of Hematology and Bone Marrow Transplantation, Wroclaw Medical University, Wroclaw, Poland; ^4^ Department of Hematology, Hospital in Gorzow Wlkp, Gorzow Wlkp, Poland; ^5^ Institute of Hematology and Transfusiology, Warsaw, Poland; ^6^ Experimental Hematooncology Department, Medical University of Lublin and Hematology Department, St John's Cancer Center in Lublin, Lublin, Poland; ^7^ Department of Internal Medicine and Hematology, Stanisław Staszic Specialist Hospital, Piła, Poland; ^8^ Department of Hematology, 109 Military Hospital, Szczecin, Poland; ^9^ Researchers of Polish Myeloma Consortium; ^10^ University of Chicago, Chicago, IL, USA; ^11^ Institute of Chemical Technology and Engineering, Poznan University of Technology, Poznan, Poland

**Keywords:** multiple myeloma, bortezomib, label-free proteomics, iTRAQ, thioredoxin

## Abstract

Identifying biomarkers of the resistance in multiple myeloma (MM) is a key research challenge. We aimed to identify proteins that differentiate plasma cells in patients with refractory/relapsed MM (RRMM) who achieved at least very good partial response (VGPR) and in those with reduced response to PAD chemotherapy (bortezomib, doxorubicin and dexamethasone). Comparative proteomic analysis was conducted on pretreatment plasma cells from 77 proteasome inhibitor naïve patients treated subsequently with PAD due to RRMM. To increase data confidence we used two independent proteomic platforms: isobaric Tags for Relative and Absolute Quantitation (iTRAQ) and label free (LF). Proteins were considered as differentially expressed when their accumulation between groups differed by at least 50% in iTRAQ and LF. The proteomic signature revealed 118 proteins (35 up-regulated and 83 down-regulated in ≥ VGPR group). Proteins were classified into four classes: (1) involved in proteasome function; (2) involved in the response to oxidative stress; (3) related to defense response; and (4) regulating the apoptotic process. We confirmed the differential expression of proteasome activator complex subunit 1 (PSME1) by enzyme-linked immunosorbent assay. Increased expression of proteasomes and proteins involved in protection from oxidative stress (eg., TXN, TXNDC5) plays a major role in bortezomib resistance.

## INTRODUCTION

Understanding the biology of multiple myeloma (MM) and identifying drug-resistance biomarkers are key research challenges that may facilitate the development of individualized treatment. Attempts to establish algorithms of therapy based on pretreatment genetic data have been proposed (e.g., Stratification of Myeloma and Risk-adapted Therapy [SMART] guidelines). These algorithms are based on the de-escalation of treatment in low-risk patients rather than the choice of a particular drug or drug combination [1]. The insightful analysis of the data points obtained for the biochemical pathways responsible for cellular processes, e.g., drug- resistance, may pave the way to truly individualized treatment. The goal of this study was to identify the biomarkers and signaling pathways that differentiate the plasma cells (PCs) of patients who achieved very good partial response (VGPR) or complete response (CR) from those with lower responses using the comparative proteomic profiling of pretreatment PCs from patients with refractory/relapsed MM (RRMM) subsequently treated with bortezomib, doxorubicin, and dexamethasone (PAD) chemotherapy. VGPR was defined according to International Myeloma Working Group (IMWG) as at least 90% disease reduction, while CR as a disappearance of monoclonal protein in both electrophoresis and immunofixation [2]. Additionally, we aimed to expand the recently published [3] catalog of proteins and pathways involved in resistance to bortezomib-based therapy in newly diagnosed MM (NDMM).

## RESULTS

**Figure 1:**
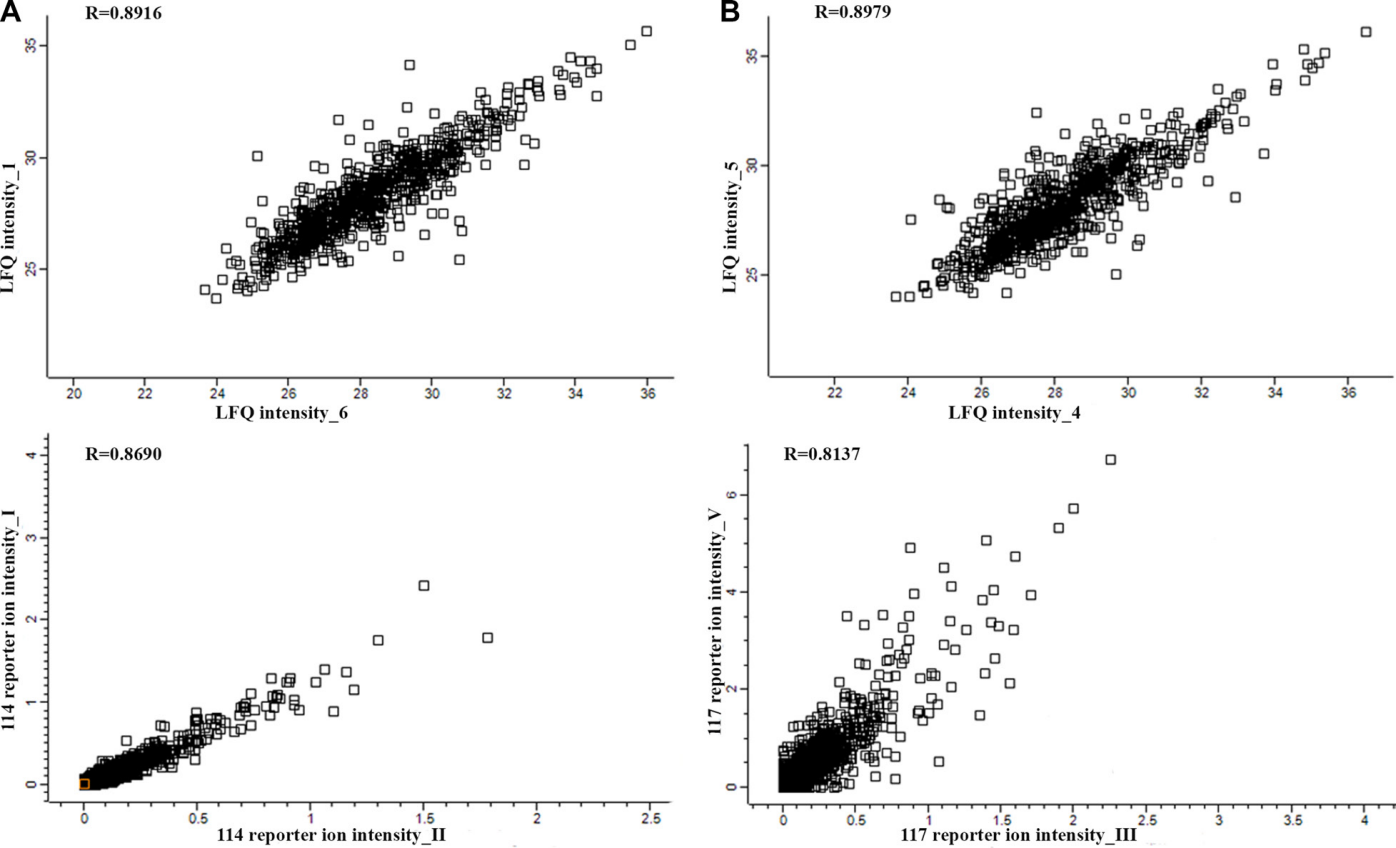
Representative correlation plots comparing the LFQ intensities and iTRAQ reporter ion intensities of two biological replications for the CR/VGPR (A) and < VGPR (B) groups The Pearson correlation coefficient is provided for each plot.

PC proteins were collected from 77 patients, digested with trypsin, and analyzed using label-free (LF) and isobaric Tags for Relative and Absolute Quantitation (iTRAQ)-based approaches. In each approach, 77 samples were prepared for digestion in duplicate. Then, each prepared sample was injected into the liquid chromatography (LC) system in duplicate in a random order. iTRAQ tag-labeled samples were analyzed using two different systems—LC matrix-assisted laser desorption/ionization tandem mass spectrometry (LC-MALDI-MS/MS) and LC electrospray ionization MS/MS (LC-ESI-MS/MS)—and the obtained data were compared. The reproducibility of the technical and biological replicates analyzed by LC-ESI-MS/MS was assessed by scatter plotting, and the correlation coefficient was determined based on the LF quantification (LFQ) intensities or iTRAQ reporter ion intensities for both experimental groups. Typical scatter plots comparing the LFQ intensities and iTRAQ reporter ion intensities are presented in Figure 1 and exhibit very good correlation among the biological replicates. Correlation analysis of the LFQ signal intensities among all biological replications gave Pearson coefficients between 0.76 and 0.96 (0.87 ± 0.09 for the CR/VGPR group; 0.81 ± 0.1 for the <VGPR group). The correlation analysis of the LFQ signal intensities between the technical replications revealed Pearson coefficients between 0.9 and 0.99 (0.94 ± 0.05; mean ± standard deviation [SD]). The correlation analysis of the iTRAQ reporter ion intensities provided Pearson coefficients between 0.72 and 0.96 (0.83 ± 0.13; mean ± SD) for both experimental groups. These results indicated that the sample replicates had a high degree of reproducibility. The Proteome Discoverer (PD) analysis revealed that the percentage of overlap between the duplicate injections exceeded 90% at the protein level. At the protein level, the percentage overlap between biological replicates from the same experimental group was 81%.

**Figure 2:**
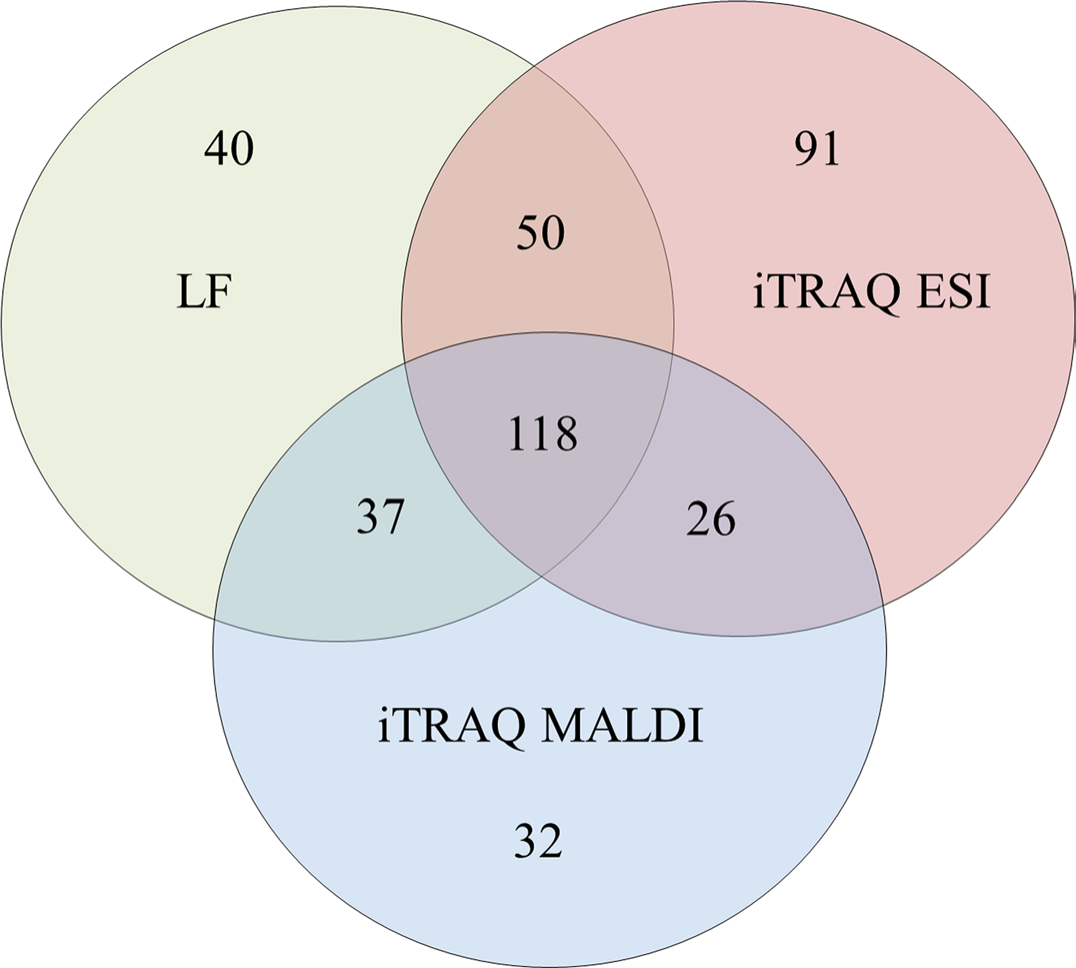
A Venn diagram comparing the results from the LF and iTRAQ (ESI and MALDI) techniques The numbers indicate differential proteins identified with two peptides using each approach.

The data derived from the LF approach were analyzed using MaxQuant (MQ) [4, 5] software. As a result, 2915 proteins were identified with a false discovery rate of 1% (FDR). Among these proteins, 2204 proteins were identified with a minimum of 2 peptides. iTRAQ data were analyzed using MQ (LC-ESI-MS/MS data) and ProteinScape (PS) (LC-MALDI-MS/MS data). In total, 2664 and 1349 proteins were identified with one peptide, and 1389 and 753 were identified with a minimum of 2 labeled peptides and 1% FDR using MQ and PS, respectively. A protein was considered to be differentially expressed if the difference was statistically significant (*p* < 0.05) and the minimum fold change was ± 1.5. Only proteins identified with a minimum of 2 peptides were considered significant. Quantitative analysis in MQ identified 245 and 285 differential proteins derived from the LF and iTRAQ approach (LC-ESI-MS/MS data), respectively. Quantitative analysis performed in PS (LC-MALDI-MS/MS data) revealed 213 differentially expressed proteins. Only 118 proteins identified by all methods and software were considered as differentially expressed between two experimental groups; these proteins are presented in Supplementary Table S1. The results obtained for LF and both iTRAQ techniques are compared in Figure 2. Among the differentially expressed proteins, 35 proteins were down-regulated, and 83 proteins were up-regulated in samples from <VGPR patients.

We used the Database for Annotation, Visualization and Integrated Discovery (DAVID) [6] and Protein Analysis Through Evolutionary Relationships (PANTHER) [7, 8] tools to identify enriched functional gene ontology (GO) annotations in the 118 differentially expressed proteins. The data were classified based on their respective molecular functions, biological processes and physiological pathways. Our analysis revealed that more than half of the proteins that were differentially expressed in CR/VGPR and < VGPR patients could be classified into four classes according to GO annotations: proteins involved in proteasome function and related to protein folding and the endoplasmic reticulum (ER) unfolded protein response (UPR) (16 proteins); proteins involved in the response to oxidative stress and cell redox homeostasis (14 proteins); proteins regulating apoptotic process and programmed cell death (21 proteins); and inflammatory and defense response proteins (16 proteins) (Table 1). Some proteins were assigned to more than one class.

**Table 1:** Comparison of the abundances of four protein classes overrepresented among differentially expressed proteins in CR/VGPR and < VGPR patients

Gene	Protein name	Fold change < VGPR vs CR/VGPR	*P* value
**Proteasome, protein folding and ER UPR**			
PSME1	Proteasome activator complex subunit 1	3.89	1.39E-04
PSME2	Proteasome activator complex subunit 2	2.42	4.31E-02
PSMA5	Proteasome subunit alpha type-5	1.96	1.85E-02
PSMA7	Proteasome subunit alpha type-7	2.03	1.28E-02
PSMB8	Proteasome subunit beta type-8	2.08	1.83E-02
PSMB2	Proteasome subunit beta type-2	1.92	1.99E-02
PSMD1	26S proteasome non-ATPase regulatory subunit 1	2.55	4.78E-02
PSMD11	26S proteasome non-ATPase regulatory subunit 11	2.23	1.83E-02
HSPA9	Stress-70 protein, mitochondrial	3.06	1.37E-05
STIP1	Stress-induced-phosphoprotein 1	1.89	4.47E-02
NPM1	Nucleophosmin	1.75	4.05E-03
GANAB	Neutral alpha-glucosidase AB	2.18	6.45E-04
HSPA6	Heat shock 70 kDa protein	2.56	3.92E-02
CACYBP	Calcyclin-binding protein	3.23	3.50E-02
PDIA6	Protein disulfide-isomerase A6	2.04	4.16E-03
HSP90AB1	Heat shock protein HSP 90-beta	1.53	1.29E-02
**Response to oxidative stress and cell redox homeostasis**			
TXN	Thioredoxin	2.91	5.92E-03
TXNDC5	Thioredoxin domain-containing protein 5	1.74	4.31E-02
PRDX3	Thioredoxin-dependent peroxide reductase	1.77	6.24E-03
TXNL1	Thioredoxin-like protein 1	2.82	4.59E-02
MPO	Myeloperoxidase	0.15	2.40E-05
PRDX2	Peroxiredoxin-2	1.60	1.99E-02
PRDX5	Peroxiredoxin-5	2.22	3.11E-05
PRDX6	Peroxiredoxin-6	1.61	4.91E-02
CAT	Catalase	0.31	4.72E-07
APEX1	DNA-(apurinic or apyrimidinic site) lyase	2.93	4.32E-02
GSTP1	Glutathione S-transferase P	0.53	9.68E-03
GLO1	Lactoylglutathione lyase	2.73	4.27E-02
NONO	Non-POU domain-containing octamer-binding protein	2.21	5.28E-03
S100A9	Protein S100-A9	0.33	8.35E-04
**Regulation of apoptotic process and programmed cell death**			
YWHAE	14–3-3 protein epsilon	1.69	2.23E-03
ANXA1	Annexin A1	0.62	4.77E-02
ANXA6	Annexin A6	0.33	7.54E-04
EIF5A	Eukaryotic translation initiation factor 5A-1	1.68	1.28E-03
LGALS1	Galectin-1	0.64	4.31E-02
HNRNPL	Heterogeneous nuclear ribonucleoprotein L	1.60	1.28E-02
HNRNPM	Heterogeneous nuclear ribonucleoprotein M	2.24	9.12E-03
MIF	Macrophage migration inhibitory factor	3.30	4.19E-05
NCL	Nucleolin	1.67	2.59E-03
NPM1	Nucleophosmin	1.75	4.05E-03
PLEC	Plectin	0.27	4.57E-03
LMNA	Prelamin-A/C	0.49	6.78E-03
PDCD4	Programmed cell death protein 4	1.91	3.26E-02
PDCD5	Programmed cell death protein 5	1.57	3.58E-02
PDCD6	Programmed cell death protein 6	2.47	1.05E-01
PDCD6IP	Programmed cell death 6-interacting protein	2.14	1.18E-02
TXNDC5	Thioredoxin domain-containing protein 5	1.74	4.31E-02
PRDX3	Thioredoxin-dependent peroxide reductase	1.77	6.24E-03
TXNL1	Thioredoxin-like protein 1	2.82	4.59E-02
VIM	Vimentin	0.58	3.94E-04
VHL	Von Hippel-Lindau disease tumor suppressor	1.99	2.42E-02
**Inflammatory and defense response**			
CTSB	Cathepsin B	0.38	4.25E-02
ISG20	Interferon-stimulated gene 20 kDa protein	0.66	3.05E-02
ANXA1	Annexin A1	0.62	4.77E-02
ANXA6	Annexin A6	0.33	7.54E-04
HMGB2	High mobility group protein B2	0.42	2.48E-06
HMGB1	High mobility group protein B1	0.65	4.07E-03
LSP1	Lymphocyte-specific protein 1	0.56	2.12E-05
MIF	Macrophage migration inhibitory factor	3.30	4.19E-05
PRTN3	Myeloblastin	0.61	1.58E-02
MPO	Myeloperoxidase	0.15	2.40E-05
DEFA3	Neutrophil defensin 3	0.21	2.18E-04
ELANE	Neutrophil elastase	0.25	4.79E-06
S100A8	Protein S100-A8	0.33	8.35E-04
S100A9	Protein S100-A9	0.33	1.28E-03
STIP	Stress-induced-phosphoprotein 1	0.57	4.47E-02
CD74	HLA class II histocompatibility antigen gamma chain	2.12	4.00E-02

This table provides the protein names, gene names, *t*-test *p*-values and calculated fold changes for both analyzed patient groups.

**Figure 3:**
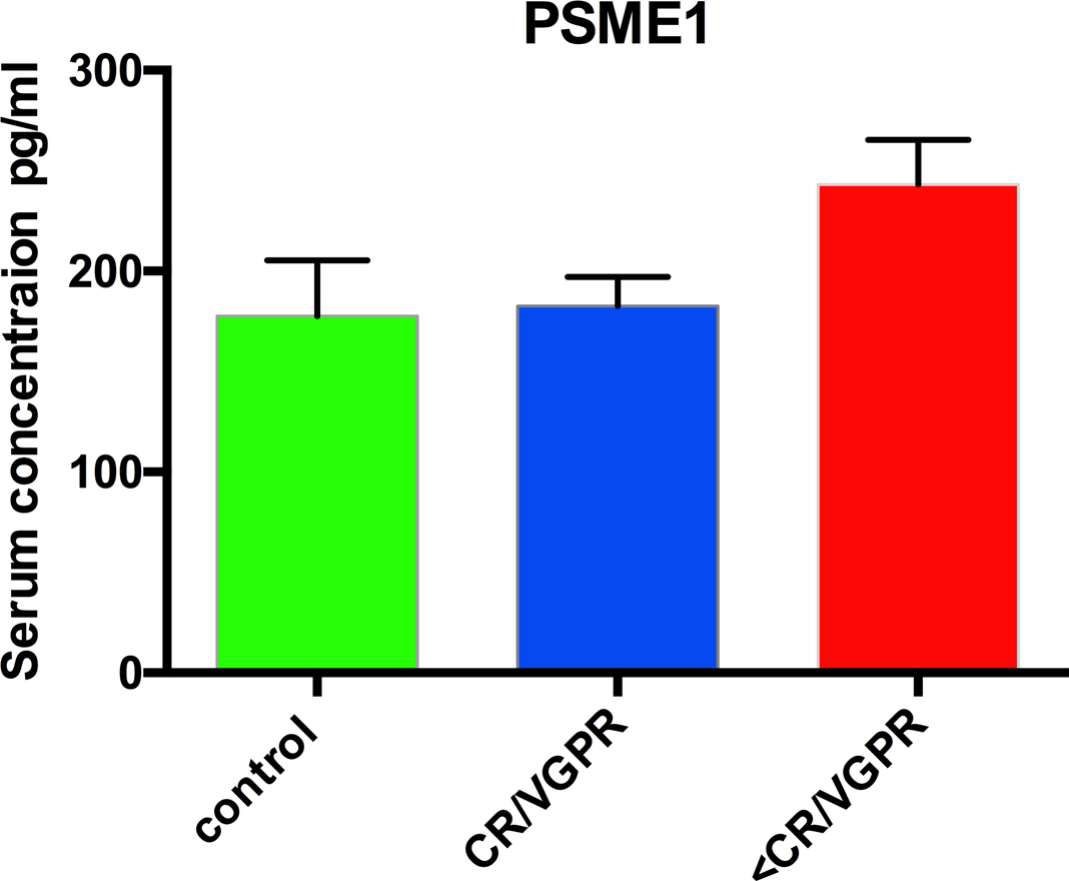
Serum concentrations of PSME1 in patients who achieved CR/VGPR (*n* = 16) to the PAD regimen vs. patients with lower response (<VGPR, *n* = 16) The controls were healthy subjects (*n* = 6). The results are presented as the mean ± standard error of the mean (SEM).

All proteins that represent proteasome proteins and molecules related to protein folding, exhibited up-regulation in <VGPR patients compared with those in CR/VGPR patients (Table 1). The accumulation of proteasome activator complex subunit 1 (PSME1) and PSME2 increased by 3.89- and 2.42-fold in <VGPR patients. The altered abundance of PSME1 was confirmed by enzyme-linked immunosorbent assay (ELISA) (CR/VGPR group: 182.7 ± 14.37; <VGPR group: 243.2 ± 22.22 pg/ml; *p* < 0.05) (Figure 3). Eight proteins related to the proteasomal structure or function were altered, and all were up-regulated in <VGPR patients. In addition, calcyclin-binding protein, which is involved in calcium-dependent ubiquitination and subsequent proteasomal degradation, is increased in <VGPR patients (fold change: 3.23). The relative amounts of proteins involved in protein folding, including heat shock protein 90 (HSP90), HSPA9, stress-induced-phosphoprotein 1, nucleophosmin and protein disulfide-isomerase, were similarly increased in <VGPR patients.

A second set of identified differential proteins included proteins that were involved in the response to oxidative stress and cellular redox homeostasis (Table 1). Compared with CR/VGPR patients, the relative abundances of thioredoxin (TXN), thioredoxin domain-containing protein 5 (TXNDC5), thioredoxin-dependent peroxide reductase and thioredoxin-like protein 1 were increased in the <VGPR group (fold changes of 2.91, 1.74, 1.77 and 2.82, respectively). In addition, three peroxiredoxins—PRDX2, PRDX5 and PRDX6—were up- regulated in <VGPR patients (fold changes of 1.6, 2.21 and 1.61, respectively). In contrast, the levels of catalase, myeloperoxidase and glutathione S-transferase P were decreased in <VGPR compared with the levels in the CR/VGPR patients (fold changes of 0.31, 0.15 and 0.53, respectively).

A third set of differential proteins was related to the regulation of apoptotic processes and programmed cell death. Two annexins, A1 and A6, and vimentin were down-regulated in <VGPR patients (fold changes of 0.32, 0.33 and 0.58, respectively). In contrast, four programmed cell death proteins were increased in <VGPR patients.

The last class of differential proteins included proteins involved in inflammatory and defense response. All of these proteins were down-regulated in <VGPR compared with CR/VGPR patients, except macrophage migration inhibitory factor and human leukocyte antigen (HLA) class II histocompatibility antigen, which were increased in <VGPR patients (fold changes of 3.3 and 2.12, respectively).

Ribosomal proteins were identified among the remaining differential proteins, and all of them were increased in <VGPR patients (Supplementary Table S1). In addition other translation proteins, including three elongation factors and valine tRNA ligase, were up-regulated in <VGPR patients compared with those of CR/VGPR patients. Nucleic acid-binding proteins constitute an additional group of proteins that exhibited increased accumulation in the <VGPR group. The relative abundances of prohibitin and prohibitin-2, which are involved in DNA synthesis, were increased in <VGPR patients compared with CR/VGPR patients (fold changes of 2.84 and 2.48, respectively). Similarly, we observed an abundance of histones H2A, H3.2 and H4 (fold changes of 1.76, 3.92 and 1.69). In addition, three proteins involved in iron binding and iron homeostasis were altered. However, compared with the CR/VGPR patients, the relative abundances of ferritin light and heavy chains and lactotransferrin were decreased in <VGPR patients (fold changes of 0.31, 0.26 and 0.12, respectively).

## DISCUSSION

This study presents the first proteomic analysis of the MM PCs of patients with RR disease that were subsequently treated with a proteasome inhibitor. Proteomic signatures differentiating patients who achieved at least VGPR and those with a lower response to bortezomib-based chemotherapy were assessed using samples from 77 patients. To increase the specificity of our results, we only analyzed those samples that contained at least 90% PCs. For further studies, we used proteins detected by at least two peptides with < 1% FDR. Because validation studies in high-throughput proteomic studies are challenging, we performed our analysis using two different independent proteomic platforms: LF and 4-plex iTRAQ. The LF and iTRAQ results were highly correlated (Figure 1). Additionally, the iTRAQ analysis was replicated using two different MS systems. Some differences were revealed in number of identified proteins (Figure 2). These differences may be explained by use of different search engines, PS engaged MASCOT, whereas for MQ ANDROMEDA engines were in use. We used an analogical method of proteomic analysis to that used in a previous study on PCs obtained from patients with NDMM [3].

Among the 118 proteins included in the proteomic signature, 16 proteins were involved in protein folding and the ER unfolded protein response. Bortezomib causes the unfolded protein response and, ultimately, cell apoptosis via proteasomal inhibition. Therefore, it is not surprising that these proteins were observed in the proteomic signatures predicting non-optimal response to PAD chemotherapy. Within that group, most proteins are components of the proteasome, including regulatory (PSME1, PSME2, PSMD1 and PSMD11) and catalytic components (PSMB2 and PMAB8). PSME1 and PSME2, which are increased in bortezomib-resistant patients, are a part of the 11S (or PA28) complex. Briefly, 11S replaces the 19S regulatory unit in the 26S constitutive proteasome and with slightly changes 20S, known as the i20S complex constitute immunoproteasomes, in response to gamma interferon [9, 10]. Both constitutive and immunoproteasomes are highly expressed in MM cells [11]. We suggest that the increased accumulation of proteasomes in PCs might be a mechanism of resistance to proteasome inhibitors. *In vitro* studies revealed that the overexpression of proteasome subunits (e.g., PSMB5) is a response to long exposure to bortezomib, but this phenomenon has not been demonstrated in a clinical setting [12]. Here, we report that an increased accumulation of proteasome subunits observed before PAD administration in RRMM PCs might indicate the lack of an efficient response to bortezomib-based treatment, similar to our previously presented results in NDMM patients [3]. Pretreatment proteasome overexpression could result in reduced treatment efficacy using standard doses of bortezomib, although this finding requires further study. We also demonstrated that increased PSME1 concentrations in patient sera correlate with the clinical response to bortezomib-based treatment and its accumulation in PCs. After further confirmation, this effect may be used as a biomarker to support treatment individualization. Calcyclin-binding protein was also a member of that functional group, and its expression has been correlated with resistance to chemotherapy in gastric cancer [13] and positively correlated with the expression of nestin (NES) and the prognostic 80-gene expression profiling (GEP80) model in MM [14].

The second set of proteins in the proteomic signature consisted of proteins involved in the responses to oxidative stress and cell redox homeostasis, including TXN, thioredoxin reductase (PRDX3), peroxiredoxins (PRDX2, PRDX5 and PRDX6) and TXNDC5, which act as antioxidants. All of these proteins were up-regulated in bortezomib-resistant patients. Reactive oxygen species (ROS) increase in neoplastic cells as a byproduct of hypermetabolism what induced the antioxidant pathway [15–17]. Increased TXN and PRDX3 accumulation was observed in studies on MM cell lines, suggesting that TXN inhibition may increase sensitivity to bortezomib treatment [18]. This result is consistent with our findings in patient samples. Our proteomic signature consisted of TXNDC5, which is a member of the protein disulfide isomerase family. Previously, we reported that increased TXNDC5 expression in PCs and serum correlates with worse response to bortezomib-based therapy in NDMM and RRMM [3, 19]. TXNDC5 is involved in proper folding of proteins and can also function as an electron transporter, recovering the functional isoform of other protein disulfide isomerases and serving the function of reduced glutathione [20]. TXN and TXNDC5 may act not only as biomarkers predicting resistance to bortezomib-based regimens but also as a potential target for treatment. Interestingly, we observed reduced expression of another set of antioxidant proteins: catalase (CAT) and glutathione S-transferase P, as previously observed in resistant acute myeloid leukemia cells [21].

The third group in the proteomic signature included proteins involved in the regulation of apoptotic processes and programmed cell death. The annexins A1 (ANXA1) and A2 (ANXA2) are down-regulated in resistant patients, whereas programmed cell death proteins 4, 5 and 6 (PDCD4, PDCD5 and PDCD6) are up-regulated. In *in vitro* studies, ANXA1 inhibition increases the sensitivity to steroid treatment in both dexamethasone-sensitive and resistant MM1 cell lines [22]; these findings are consistent with ours. Surprisingly, PDCDs, the tumor suppressor proteins that promote apoptosis, were down-regulated in refractory patients. PDCD4 down-regulation is associated with poor prognosis in breast cancer [23].

Among the fourth set of proteins, which are involved in inflammation and defense responses, S100A8 and S100A9 belong to the calcium-binding protein family and were down-regulated in the <VGPR group. The role of these proteins in drug resistance is well known and was previously described as being primarily based on autophagy and the activation of myeloid-derived suppressor cells (MDSCs) [24, 25].

## MATERIALS AND METHODS

### Subjects and samples

Our study protocol conformed to the Ethical Guidelines of the World Medical Association Declaration of Helsinki. Before the project commenced, appropriate approval was obtained from the Bioethical Commission of the Karol Marcinkowski University of Medical Sciences, Poznan, Poland. Informed consent was obtained from all participating individuals prior to participation in the study. Comparative proteome analysis was performed on PCs obtained from the pretreatment bone marrow of 77 proteasome inhibitor naïve patients (median age 67 (52– 75)) who qualified for PAD (bortezomib, doxorubicin, dexamethasone) chemotherapy. The first line treatment was based on thalidomide (CTD, TD or MPT). The PAD regimen was administered as a second-line treatment because of RR disease [26]. Patients received a median of 6 cycles (range 2–8) of treatment. Treatment was interrupted because of toxicity for 10 patients (G3 neuropathy and G2 infection) and disease progression (PD) (8 patients). Disease response to treatment was assessed by International Myeloma Working Group (IMWG) criteria [2]. After chemotherapy, 30 patients achieved CR or VGPR (CR/VGPR group), and 47 achieved lower responses (<VGPR group), including partial response PR (27), stable disease (SD) (2) and progressive disease (PD) (18).

### PC isolation

Bone marrow samples were obtained after patients signed informed consent and were freshly purified by negative selection (EasySep, STEMCELL Technologies, Vancouver, Canada) according to the manufacturer's protocol. PCs were frozen as pellets containing 500,000 cells in liquid nitrogen and stored until proteomic analysis.

### Protein extraction

PCs were lysed in buffer with 1 M triethylammonium bicarbonate (TEAB) and 0.1% sodium dodecyl sulfate (SDS) and automatically homogenized using a Precellys 24 homogenizer (Bertin Technologies, France) in 0.5-mL tubes pre-filled with ceramic (zirconium oxide) beads (Bertin Technologies, France). For all homogenization procedures, 20 μl of buffer was added to each 100,000 cells and processed at 6,300 rpm for 30 seconds three times. Then, the material was sonicated in a bath for three 1-minute cycles on ice and homogenized again using the Precellys 24 instrument. The homogenized suspension was centrifuged at 16,000 × *g* for 10 min at 4°C, and the supernatants were retained for analysis. The protein concentration was determined using the bicinchoninic acid assay (BCA; Pierce) method.

### LF-based proteomic approach

#### NanoLC-MS/MS analysis

Ten micrograms of PC protein was digested and prepared for analysis as previously described [27]. Each sample was prepared for digestion in duplicate. For each run, 1.5 μg of the digested protein samples was subjected to nano-LC MS/MS analysis using a Dionex UltiMate 3000 RSLC nano System and Q-Exactive Orbitrap mass spectrometer (Thermo Fisher Scientific, USA), as previously described [27]. The normalized collision energy was set to 28. Each sample was injected in duplicate at random.

#### Qualitative analysis of LF proteomic data

After each LC-MS/MS run, the raw files were qualitatively analyzed by PD version 1.4.14 (Thermo Fisher Scientific, USA). To evaluate the quality of the performed runs, the numbers of peptide spectrum matches (PSMs) and identified proteins were calculated. LC-MS/MS runs with fewer than 150,000 PSMs and fewer than 2,000 identified proteins (1% FDR) were excluded from further analysis. The identification of proteins by PD was performed using the SEQUEST engine against the UniProt Complete Proteome Set of Humans (123,619 sequences) using the following parameters: a tolerance level of 10 ppm for MS and 0.05 Da for MS/MS, and two missed cleavages were allowed. The carbamidomethylation of cysteines was set as a fixed modification, and the oxidation of methionines was allowed as a variable modification.

#### Assessment of variability/reproducibility

The technical and biological variabilities of each plasma sample from both experimental groups were estimated by scatter plot and calculated using the Pearson correlation coefficients of the LFQ intensities in Perseus. To assess the reproducibility, the percentage overlap between the protein identifications for both the technical/injection and biological replicates was calculated using PD software. The LFQ intensities derived from all of the samples evaluated in PD were subjected to statistical analysis.

### Quantitative analysis of LF proteomic data and statistical analysis

The raw files that were positively evaluated by PD were quantitatively analyzed by MQ/Perseus [4, 5] version 1.5.1.2, as previously described [27]. A protein was considered to be differentially expressed if the difference was statistically significant (*p* < 0.05), the minimum fold change was +/− 1.5, and it was identified with a minimum of 2 peptides with >99% confidence. Multivariate analyses were conducted by untargeted principal component analysis (PCA). All statistical analyses were performed using Statistica v. 10.0 software (StatSoft, Inc., www.statsoft.com) and Perseus 1.4.1.3, which is freely available from the MQ web site.

### iTRAQ-based proteomic approach

#### Protein digestion and iTRAQ labeling

Protein digestion was performed before iTRAQ labeling on 75-μg aliquots of PC proteins according to the manufacturer's instructions (AB Sciex, USA) with the following minor modifications. The proteins were overnight digested with 2 μg of sequencing-grade trypsin (Promega, Germany) at 37°C. For labeling, each iTRAQ reagent (AB Sciex, USA) was added to the respective peptide mixture for 120 min. In all of the iTRAQ experiments, all of the analyzed groups were labeled with the same iTRAQ tag as follows: 114 and 115, samples from CR/VGPR patients; 116 and 117, samples from <VGPR patients. The labeling reaction was quenched by the addition of 125 μl of Milli-Q water, and four labeled samples were then pooled into one sample according to the manufacturer's instructions. After pooling, the samples were evaporated to 50 μl by vacuum concentration to remove excess water, TEAB, and ethanol. The labeled digest was purified and fractionated on a SCX cartridge system (AB Sciex, USA) off-line. The peptides were sequentially eluted from the columns with increasing concentrations of KCl. Four fractions were collected in 87.5, 175, 350, and 500 mM KCl using 350-μl aliquots of KCl in 10 mM KH_2_PO_4_ and 25% (v/v) acetonitrile. The collected fractions were finally desalted on SPE BakerBond™ C18 Cartridges (J.T. Baker, USA) and then evaporated to 50 μl by vacuum concentration to remove the acetonitrile.

### ESI-NanoLC-MS/MS analysis

For each iTRAQ experiment, 5 μl of each respective KCl fraction was injected into the same LC-MS/MS system using the same conditions as for the LF approach with the following minor modifications. The fixed first mass was *m*/*z* 100.0. The normalized collision energy was set to 30. To reduce the interference of precursor co-fragmentation with the iTRAQ quantification, the isolation window was set to 1.2 *m*/*z*.

### MALDI off-line LC-MS/MS analysis

For each iTRAQ experiment, 5 μl of the respective KCl fraction was analyzed in duplicate. The samples from each iTRAQ experiment were subjected to nano-LC separation using an EASY-nLC Proxeon (Bruker Daltonics, Germany) coupled to a Proteineer fc II (Bruker Daltonics, Germany) fraction collector, as previously described [28]. MS/MS analyses were performed using a UltrafleXtreme MALDI-TOF/TOF (Bruker Daltonics, Germany) instrument, as previously described [28].

### Analysis of iTRAQ data

Raw files derived from ESI-LC-MS/MS were analyzed in PD version 1.4.14 (Thermo Fisher Scientific) and MQ/Perseus software version 1.5.1.2 using the same database as for the LF approach. The Percolator software integrated in the PD was used to evaluate the database search results. The obtained MALDI data were analyzed using the PS (Bruker Daltonics) database software against the UniProt database. The FDR for peptide identification in PD, MQ and PS was set at 1%. The parameters for database searching were as follows: iTRAQ 4-plex (peptide-labeled) modification and tolerance levels of 10 ppm for MS, 0.05 Da for MS/MS (ESI-LC-MS/MS), 0.3 Da for MS, and 0.5 Da for MS/MS (MALDI-LC-MS/MS). Other parameters were the same as for the LF approach. The relative peptide abundance was measured using the iTRAQ reporter ion peak area ratios. The following ratios were calculated: 114/115, 114/116, 114/117, 115/116, 115/117 and 116/117. MQ data were evaluated, and statistical analysis was performed using the Perseus software (version 1.4.1.3, Max Planck Institute of Biochemistry, Martinsried) as previously described [27]. The mean values ± SDs were calculated from the reporter peak area ratios of all labeled peptides for a given protein. Proteins with a fold change of at least 1.5 identified by MQ/Perseus and PS were considered to be differentially expressed and were then statistically analyzed.

### Assessment of variability/reproducibility and statistical analysis

The reproducibility of technical and biological replicates was assessed by scatter plotting and correlation coefficient determination based on reporter ion signals. The percentage overlap in protein identification between both technical/injection and biological replicates was calculated. Coefficient of variation (CV) values were the primary parameters used to validate the data. Proteins with variability in reporter ion signals exceeding 30% were excluded from the analysis, as described earlier [29]. Positively evaluated reporter ion intensities derived from all samples and from all iTRAQ experiments were considered in the statistical analysis, as for the LF approach.

### ELISA validation

Serum samples from patients treated with a PAD regimen were collected before the first dose of proteasome inhibitor. Samples were frozen and stored at −80°C until assessment. The assay was performed using PSME1 ELISA kits (Cusabio, USA) according to the manufacturer's instructions. Mathematical and statistical analyses were performed using Curve Expert v1.3 (Hyams Development) and PRISM. The results are presented as the mean ± standard error of the mean (SEM).

### Functional analyses of dysregulated proteins in PC samples

Only the proteins that were quantified as unique and non-redundant were used in the subsequent analyses. Proteins with a fold change of at least 1.5 that were identified as differential by both LF- and iTRAQ-based approaches were considered to be differentially expressed and were subsequently analyzed. The dysregulated proteins were chosen based on the criterion that the protein must be quantified by a minimum of two peptides with >99% confidence. Uncharacterized proteins were excluded from the analysis. The differential proteins were analyzed using the DAVID (http://david.abcc.ncifcrf.gov/) [6] and PANTHER (http://pantherdb.org/) [7] analysis tools to identify enriched functions, biological process and pathways categories [8]. Benjamini-corrected *P*-values less than 0.05 were considered significant. Pathway analysis using the DAVID tool was based on the REACTOME, KEGG pathway and PANTHER databases.

### SUPPLEmentary Table S1

Complete list of differential proteins identified in the PCs of <VGPR and CR/VGPR patients. The table provides the protein names, numbers of identified peptides, scores, MS/MS counts, sequence coverages, molecular weights, calculated mean fold changes for iTRAQ and LF approaches, SwissProt accession numbers and the GO terms of the molecular function/biological process.

## SUPPLEMENTARY MATERIALS TABLE


