# Supplementary material for: Comparative proteomic profiling of refractory/relapsed multiple myeloma reveals biomarkers involved in resistance to bortezomib-based therapy
Source: Oncotarget. 2016 Aug 4;7(35):56726–36. doi: 10.18632/oncotarget.11059 (PMC5302948; doi:10.18632/oncotarget.11059)
Supplement: Supplementary file 1 [file oncotarget-07-56726-s001.doc]

**Supplementary Table S1**

Complete list of differential proteins identified in the PCs of <VGPR and CR/VGPR patients.

| **Lp** | **Score** | **MS/MS Count** | **Unique sequence coverage [%]** | **Molecular weight [kDa]** | **Unique peptides** | **Fold change <VGPR *vs.* CR/VGPR** | **Protein IDs** | **Protein names** | **Gene names** | **GO - Molecular function/biological process** |
| --- | --- | --- | --- | --- | --- | --- | --- | --- | --- | --- |
| 1 | 131.38 | 198 | 50 | 15.388 | 2 | 3.93 | Q71DI3 | Histone H3.2 | HIST2H3A | DNA binding |
| 2 | 154.93 | 1547 | 71.5 | 28.723 | 19 | 3.89 | Q06323 | Proteasome activator complex subunit 1 | PSME1 | ubiquitin-dependent protein degradation, apoptotic process, GTPase mediated signal transduction |
| 3 | 47.983 | 43 | 51.6 | 21.995 | 7 | 3.56 | Q6FGV9 | Phosphomevalonate kinase | PMVK | phosphorylation and kinase activity, cholesterol biosynthetic process |
| 4 | 19.556 | 844 | 17.4 | 12.476 | 2 | 3.30 | I4AY87 | Macrophage migration inhibitory factor | MIF | DNA damage response, protein phosphorylation, defense response, apoptotic activity |
| 5 | 95.841 | 36 | 52.2 | 26.21 | 6 | 3.23 | Q9HB71 | Calcyclin-binding protein | CACYBP | calcium-dependent ubiquitination, proteosomal degradation |
| 6 | 323.31 | 791 | 58.7 | 113.08 | 50 | 3.21 | P09874 | Poly [ADP-ribose] polymerase 1 | PARP1 | DNA binding and damage response |
| 7 | 39.091 | 31 | 11.6 | 59.151 | 4 | 3.17 | Q8NF37 | Lysophosphatidylcholine acyltransferase 1 | LPCAT1 | calcium ion binding, lipid process |
| 8 | 209.98 | 1203 | 62.8 | 56.049 | 22 | 3.10 | B4DQG5 | Cytosol aminopeptidase | LAP3 | proteolysis |
| 9 | 2.5257 | 17 | 24.2 | 10.32 | 2 | 3.06 | P84103 | Serine/arginine-rich splicing factor 3 | SFRS3 | DNA binding |
| 10 | 245.88 | 1510 | 21.7 | 72.4 | 11 | 3.06 | V9HW84 | Stress-70 protein, mitochondrial | HSPA9 | protein folding |
| 11 | 323.31 | 206 | 66 | 35.554 | 15 | 2.93 | Q5TZP7 | DNA-(apurinic or apyrimidinic site) lyase, mitochondrial | APEX1 | response to oxidative stress, cell redox homeostasis |
| 12 | 40.691 | 495 | 65.7 | 11.737 | 8 | 2.91 | H9ZYJ2 | Thioredoxin | TXN | oxidoreductase activity, antioxidant activity, response to oxidative stress |
| 13 | 83.793 | 42 | 12.6 | 51.105 | 3 | 2.90 | Q8N684 | Cleavage and polyadenylation specificity factor subunit 7 | CPSF7 | nucleic acids binding |
| 14 | 323.31 | 324 | 78.7 | 29.804 | 15 | 2.84 | A8K401 | Prohibitin | PHB | nucleic acids binding, modulator of transcriptional activity |
| 15 | 253.26 | 877 | 40 | 52.645 | 15 | 2.82 | P31930 | Cytochrome b-c1 complex subunit 1, mitochondrial | UQCRC1 | oxidation-reduction process |
| 16 | 203.21 | 62 | 43.3 | 32.251 | 8 | 2.82 | O43396 | Thioredoxin-like protein 1 | TXNL1 | cell redox homeostasis, antioxidant activity, apoptotic process |
| 17 | 101.85 | 180 | 54.9 | 20.777 | 11 | 2.73 | X5DNM4 | Lactoylglutathione lyase | GLO1 | glutathione metabolic process, regulation of transcription |
| 18 | 132.92 | 1279 | 57.4 | 11.333 | 5 | 2.68 | C9JFR7 | Cytochrome c | CYCS | oxidation-reduction process |
| 19 | 8.6199 | 16 | 16.3 | 70.971 | 2 | 2.56 | Q53FC7 | Heat shock 70 kDa protein 6 | HSPA6 | unfolded protein binding |
| 20 | 275.29 | 101 | 35 | 105.84 | 13 | 2.55 | Q99460 | 26S proteasome non-ATPase regulatory subunit 1 | PSMD1 | ubiquitin-dependent protein degradation, |
| 21 | 323.31 | 1688 | 37.3 | 50.431 | 13 | 2.54 | B4DJV2 | Citrate synthase mitochondrial | CS | tricarboxylic acid cycle |
| 22 | 190.67 | 275 | 63.8 | 33.239 | 13 | 2.48 | J3KPX7 | Prohibitin-2 | PHB2 | nucleic acids binding, modulator of transcriptional activity |
| 23 | 17.782 | 30 | 28.3 | 21.868 | 4 | 2.47 | O75340 | Programmed cell death protein 6 | PDCD6 | DNA binding, programmed cell death |
| 24 | 322.08 | 5724 | 48.9 | 50.91 | 20 | 2.46 | Q53GL5 | Isocitrate dehydrogenase [NADP], mitochondrial | IDH2 | tricarboxylic acid cycle |
| 25 | 323.31 | 275 | 64.9 | 27.401 | 22 | 2.42 | Q9UL46 | Proteasome activator complex subunit 2 | PSME2 | ubiquitin-dependent protein degradation, apoptotic process, GTPase mediated signal transduction |
| 26 | 126.9 | 1425 | 31.2 | 47.716 | 12 | 2.42 | P23526 | Adenosylhomocysteinase | AHCY | L-homocysteine biosynthesis, response to hypoxia |
| 27 | 323.31 | 1763 | 37.7 | 82.704 | 25 | 2.25 | P13010 | X-ray repair cross-complementing protein 5 | XRCC5 | damaged DNA binding and repair |
| 28 | 137.2 | 1155 | 43.2 | 77.569 | 27 | 2.24 | A0A087X0X3 | Heterogeneous nuclear ribonucleoprotein M | HNRNPM | DNA binding , regulation of apoptotic process |
| 29 | 132.26 | 137 | 58.1 | 47.463 | 17 | 2.23 | O00231 | 26S proteasome non-ATPase regulatory subunit 11 | PSMD11 | ubiquitin-dependent protein degradation, |
| 30 | 189.06 | 1309 | 42.5 | 75.21 | 24 | 2.23 | P49748 | Very long-chain specific acyl-CoA dehydrogenase, mitochondrial | ACADVL | oxidation-reduction process |
| 31 | 323.31 | 3428 | 53.2 | 59.75 | 26 | 2.23 | V9HW26 | ATP synthase subunit alpha, mitochondrial | ATP5A1 | ATP binding, ATP biosynthetic process |
| 32 | 86.909 | 1335 | 47.5 | 17.031 | 6 | 2.22 | P30044 | Peroxiredoxin-5, mitochondrial | PRDX5 | oxidoreductase activity, response to oxidative stress |
| 33 | 253.4 | 1238 | 43.5 | 54.287 | 7 | 2.21 | Q15233 | Non-POU domain-containing octamer-binding protein | NONO | response to oxidative stress, RNA splicing factor |
| 34 | 323.31 | 1007 | 46.8 | 72.691 | 15 | 2.14 | A0A024QZ30 | Succinate dehydrogenase [ubiquinone] flavoprotein subunit, mitochondrial | SDHA |  |
| 35 | 191.57 | 141 | 43.4 | 96.078 | 27 | 2.14 | Q8WUM4 | Programmed cell death 6-interacting protein | PDCD6IP | calcium-dependent protein binding, programmed cell death |
| 36 | 323.31 | 3294 | 67.7 | 49.541 | 27 | 2.13 | P49411 | Elongation factor Tu, mitochondrial | TUFM | translational elongation |
| 37 | 55.127 | 291 | 52.6 | 40.926 | 2 | 2.12 | P04233 | HLA class II histocompatibility antigen gamma chain | CD74 | antigen processing and presentation, immune response |
| 38 | 192.78 | 1509 | 50.7 | 69.842 | 13 | 2.10 | P12956 | X-ray repair cross-complementing protein 6 | XRCC6 | damaged DNA binding and repair |
| 39 | 87.04 | 51 | 26.8 | 29.696 | 4 | 2.08 | Q6FHU0 | Proteasome subunit beta type-8 | PSMB8 | ubiquitin-dependent protein degradation, apoptotic process, GTPase mediated signal transduction |
| 40 | 173.02 | 790 | 83.9 | 26.923 | 7 | 2.06 | Q99714 | 3-hydroxyacyl-CoA dehydrogenase type-2 | HSD17B10 | oxidation-reduction process |
| 41 | 323.31 | 1670 | 54.3 | 48.121 | 15 | 2.04 | Q15084 | Protein disulfide-isomerase A6 | PDIA6 | endoplasmic reticulum unfolded protein response, inhibits aggregation of misfolded proteins. |
| 42 | 181.49 | 121 | 58.8 | 26.713 | 7 | 2.03 | O14818 | Proteasome subunit alpha type-7 | PSMA7 | ubiquitin-dependent protein degradation, apoptotic process, GTPase mediated signal transduction |
| 43 | 95.505 | 675 | 59.5 | 32.118 | 11 | 2.03 | P00491 | Purine nucleoside phosphorylase | PNP | purine metabolism |
| 44 | 1.5481 | 18 | 5.3 | 15.219 | 2 | 1.99 | A0A024R2F2 | Von Hippel-Lindau disease tumor suppressor | VHL | apoptotic process |
| 45 | 92.239 | 2119 | 71.9 | 32.642 | 2 | 1.97 | Q32Q12 | Nucleoside diphosphate kinase | NME1 | phosphorylation, nucleotide binding |
| 46 | 323.31 | 3404 | 49.6 | 192.06 | 45 | 1.96 | A0A087WVQ6 | Clathrin heavy chain 1 | CLTC | nucleic adids binding, antigen processing and presentation |
| 47 | 177.81 | 154 | 56.4 | 26.425 | 8 | 1.96 | P28066 | Proteasome subunit alpha type-5 | PSMA5 | ubiquitin-dependent protein degradation, apoptotic process, GTPase mediated signal transduction |
| 48 | 323.31 | 1774 | 45.2 | 140.47 | 24 | 1.93 | P26640 | Valine--tRNA ligase | VARS | protein translation |
| 49 | 46.727 | 56 | 37.3 | 22.836 | 5 | 1.92 | P49721 | Proteasome subunit beta type-2 | PSMB2 | ubiquitin-dependent protein degradation, apoptotic process, GTPase mediated signal transduction |
| 50 | 287.99 | 1304 | 60.8 | 30.772 | 13 | 1.92 | P21796 | Voltage-dependent anion-selective channel protein 1 | VDAC1 | voltage-gated anion channel activity, apoptotic process |
| 51 | 183.67 | 57 | 36.7 | 50.228 | 7 | 1.91 | B4DKX4 | Programmed cell death protein 4 | PDCD4 | DNA binding, programmed cell death |
| 52 | 256.59 | 1016 | 39.9 | 80.439 | 8 | 1.91 | H3BLZ8 | Probable ATP-dependent RNA helicase DDX17 | DDX17 | nucleic acids binding |
| 53 | 21.745 | 107 | 23.4 | 62.639 | 11 | 1.89 | A8K690 | Stress-induced-phosphoprotein 1 | STIP1 | defense response, ER UPR |
| 54 | 323.31 | 4824 | 5.7 | 56.559 | 2 | 1.86 | P06576 | ATP synthase subunit beta, mitochondrial | ATP5B | ATP binding, ATP biosynthetic process |
| 55 | 285.17 | 3076 | 3.6 | 46.566 | 2 | 1.83 | Q5JP53 | Tubulin beta chain | TUBB | GTPase activity, constituent of cytoskeleton, protein folding |
| 56 | 247.97 | 1013 | 70.5 | 14.515 | 8 | 1.79 | P25398 | 40S ribosomal protein S12 | RPS12 | structural constituent of ribosome, translation |
| 57 | 104.28 | 680 | 52 | 27.692 | 7 | 1.77 | P30048 | Thioredoxin-dependent peroxide reductase, mitochondrial | PRDX3 | apoptotic process, antioxidant activity |
| 58 | 323.31 | 2890 | 76.1 | 50.151 | 2 | 1.76 | P68363 | Tubulin alpha-1B chain | TUBA1B | GTPase activity, constituent of cytoskeleton, protein folding |
| 59 | 204.61 | 6869 | 53.9 | 13.509 | 3 | 1.76 | Q71UI9 | Histone H2A.V | H2AFV H2AFZ | nucleic acids binding |
| 60 | 323.31 | 1333 | 6.4 | 33.515 | 8 | 1.75 | P06748 | Nucleophosmin | NPM1 | unfolded protein binding, DNA repair, apoptotic process |
| 61 | 323.31 | 11317 | 88.2 | 40.369 | 28 | 1.74 | Q86UY0 | Thioredoxin domain-containing protein 5 | TXNDC5 | cell redox homeostasis, antioxidant activity, apoptotic process |
| 62 | 208.76 | 1213 | 53.4 | 29.505 | 10 | 1.73 | C9J9K3 | 40S ribosomal protein SA | RPSA | structural constituent of ribosome, translation |
| 63 | 323.31 | 1380 | 46.4 | 63.146 | 2 | 1.72 | P06744 | Glucose-6-phosphate isomerase | GPI | glycolysis |
| 64 | 159.58 | 7060 | 68 | 11.367 | 11 | 1.70 | B2R4R0 | Histone H4 | HIST1H4H | nucleic acids binding |
| 65 | 190.04 | 1915 | 33.5 | 31.969 | 8 | 1.70 | Q0QF37 | Malate dehydrogenase, mitochondrial | MDH2 | tricarboxylic acid cycle, oxidoreductase activity |
| 66 | 109.79 | 1861 | 61.3 | 26.689 | 14 | 1.69 | P23396 | 40S ribosomal protein S3 | RPS3 | structural constituent of ribosome, translation |
| 67 | 323.31 | 2934 | 42.4 | 29.174 | 10 | 1.69 | P62258 | 14-3-3 protein epsilon | YWHAE | protein binding, apoptotic process, GTPase mediated signal transduction |
| 68 | 317.45 | 1164 | 66.2 | 16.832 | 8 | 1.68 | P63241 | Eukaryotic translation initiation factor 5A-1 | EIF5A | protein biosynthesis, stress response, apoptotic process, |
| 69 | 323.31 | 1553 | 24.8 | 68.441 | 11 | 1.67 | P19338 | Nucleolin | NCL | calcium ion binding, apoptotic process |
| 70 | 102.1 | 672 | 39 | 22.391 | 5 | 1.64 | M0R0F0 | 40S ribosomal protein S5 | RPS5 | structural constituent of ribosome, translation |
| 71 | 25.423 | 758 | 49.8 | 16.06 | 8 | 1.64 | B0ZBD0 | 40S ribosomal protein S19 | RPS19 | structural constituent of ribosome, translation |
| 72 | 135.57 | 1485 | 66.5 | 25.035 | 13 | 1.61 | P30041 | Peroxiredoxin-6 | PRDX6 | oxidoreductase activity, response to oxidative stress |
| 73 | 315.32 | 674 | 49.3 | 61.926 | 3 | 1.60 | P14866 | Heterogeneous nuclear ribonucleoprotein L | HNRNPL | DNA binding , regulation of apoptotic process |
| 74 | 109.76 | 1233 | 57.6 | 21.892 | 9 | 1.60 | P32119 | Peroxiredoxin-2 | PRDX2 | oxidoreductase activity, response to oxidative stress |
| 75 | 33.152 | 1087 | 47 | 17.222 | 6 | 1.57 | P62277 | 40S ribosomal protein S13 | RPS13 | structural constituent of ribosome, translation |
| 76 | 11.376 | 17 | 40.2 | 10.004 | 2 | 1.57 | K7EQA1 | Programmed cell death protein 5 | PDCD5 | DNA binding, programmed cell death |
| 77 | 323.31 | 1521 | 64.7 | 50.663 | 12 | 1.57 | P50395 | Rab GDP dissociation inhibitor beta | GDI2 | GTPase mediated signal transduction, oxidoreductase activity |
| 78 | 323.31 | 3085 | 68.7 | 36.688 | 17 | 1.56 | V9HWB9 | L-lactate dehydrogenase | LDHA | oxidoreductase activity |
| 79 | 216.75 | 1371 | 70.1 | 26.922 | 10 | 1.55 | Q5SRT3 | Chloride intracellular channel protein 1 | CLIC1 | regulation of cell cycle, chloride channel activity |
| 80 | 143.44 | 909 | 53.6 | 21.863 | 3 | 1.55 | P32969 | 60S ribosomal protein L9 | RPL9 | structural constituent of ribosome, translation |
| 81 | 76.152 | 1227 | 43.2 | 29.975 | 9 | 1.54 | Q6NXR8 | 40S ribosomal protein S3a | RPS3A | structural constituent of ribosome, translation |
| 82 | 323.31 | 2002 | 61.1 | 50.118 | 16 | 1.54 | P26641 | Elongation factor 1-gamma | EEF1G | translation elongation factor activity |
| 83 | 323.31 | 4339 | 61.5 | 79.194 | 13 | 1.53 | A0A024RD80 | Heat shock protein HSP 90-beta | HSP90AB1 | GTPase mediated signal transduction |
| 84 | 30.126 | 356 | 76.8 | 20.825 | 6 | 0.67 | P61224 | Ras-related protein Rap-1b | RAP1B | blood coagulation, GTPase mediated signal transduction |
| 85 | 42.665 | 391 | 53.6 | 53.6 | 8 | 0.66 | Q96AZ6 | Interferon-stimulated gene 20 kDa protein | ISG20 | defense response |
| 86 | 263.68 | 680 | 31.2 | 24.893 | 8 | 0.65 | P09429 | High mobility group protein B1 | HMGB1 | defense response, DNA binding |
| 87 | 18.438 | 500 | 8 | 39.811 | 3 | 0.64 | P13473 | Lysosome-associated membrane glycoprotein 2 | LAMP2 | lysosomal protein |
| 88 | 49.93 | 648 | 57 | 14.716 | 6 | 0.64 | P09382 | Galectin-1 | LGALS1 | signal transducer activity, apoptotic process |
| 89 | 133.76 | 106 | 36.8 | 54.336 | 2 | 0.62 | P08133 | Annexin A6;Annexin | ANXA6 | calcium ion binding, inflammatory response, Apoptosis |
| 90 | 196.37 | 691 | 60.5 | 20.457 | 3 | 0.62 | J3QRS3 | Myosin regulatory light chain | MYL12A | calcium ion binding, protein targeting to plasma membrane |
| 91 | 22.86 | 46 | 18.1 | 23.611 | 4 | 0.61 | U3KPS2 | Myeloblastin | PRTN3 | blood coagulation, response to stimulus |
| 92 | 323.31 | 7871 | 56.2 | 53.651 | 29 | 0.58 | P08670 | Vimentin | VIM | apoptotic process, regulation of gene expression |
| 93 | 10.961 | 75 | 43.1 | 20.409 | 6 | 0.58 | P61106 | Ras-related protein Rab-14 | RAB14 | GTP binding, transport |
| 94 | 197.17 | 354 | 16.4 | 37.191 | 4 | 0.56 | P33241 | Lymphocyte-specific protein 1 | LSP1 | signal transducer activity, defense response |
| 95 | 154.55 | 1008 | 60.5 | 23.356 | 5 | 0.53 | P09211 | Glutathione S-transferase P | GSTP1 | oxidoreductase activity, glutathion activity, response to oxidative stress |
| 96 | 20.973 | 578 | 56.2 | 27.174 | 2 | 0.53 | P06753 | Tropomyosin alpha-3 chain | TPM3 | actin binding motor protein |
| 97 | 117.23 | 935 | 35.8 | 74.139 | 19 | 0.49 | P02545 | Prelamin-A/C | LMNA | apoptotic process, endoplasmic reticulum unfolded protein response |
| 98 | 301.42 | 1085 | 41.6 | 16.837 | 6 | 0.49 | B4DJ51 | Calmodulin | CALM1 | calcium ion binding, calcium-mediated signaling |
| 99 | 51.989 | 879 | 20.6 | 58.14 | 9 | 0.47 | Q53FJ5 | Prosaposin | PSAP | lipid metabolic process, lysosome |
| 100 | 323.31 | 3701 | 36.8 | 226.53 | 68 | 0.47 | A0A024R1N1 | Myosin-9 | MYH9 | motor protein, actin-binding |
| 101 | 45.588 | 437 | 58.5 | 17.936 | 7 | 0.46 | F5H3P3 | Rho GDP-dissociation inhibitor 2 | ARHGDIB | GTPase mediated signal transduction |
| 102 | 115.24 | 1057 | 53.6 | 70.289 | 27 | 0.46 | Q53FI1 | Plastin-2 | LCP1 | calcium ion binding, actin binding |
| 103 | 38.848 | 771 | 18.8 | 23.904 | 5 | 0.42 | P26583 | High mobility group protein B2 | HMGB2 | defense response, DNA binding |
| 104 | 38.149 | 184 | 29.4 | 28.837 | 3 | 0.38 | A0A024R374 | Cathepsin B | CTSB | defense response |
| 105 | 323.31 | 1196 | 34.7 | 269.76 | 30 | 0.34 | Q9Y490 | Talin-1 | TLN1 | integrin binding, blood coagulation |
| 106 | 247.9 | 1517 | 90.4 | 13.242 | 10 | 0.33 | P06702 | Protein S100-A9 | S100A9 | antioxidant activity, calcium ion binding, inflammatory response |
| 107 | 59.744 | 1064 | 54.8 | 10.834 | 10 | 0.33 | P05109 | Protein S100-A8 | S100A8 | calcium ion binding, defense response |
| 108 | 58.453 | 544 | 47.4 | 38.714 | 14 | 0.33 | P04083 | Annexin A1 | ANXA1 | calcium ion binding, inflammatory response, apoptosis |
| 109 | 142.61 | 1965 | 75.4 | 20.019 | 14 | 0.32 | P02792 | Ferritin light chain | FTL | cellular iron ion homeostasis |
| 110 | 125.92 | 430 | 39.5 | 59.755 | 17 | 0.31 | P04040 | Catalase | CAT | antioxidant activity |
| 111 | 323.31 | 1764 | 42 | 276.55 | 36 | 0.28 | Q5HY54 | Filamin-A | FLNA | integrin signaling pathway |
| 112 | 323.31 | 1022 | 13.7 | 531.78 | 62 | 0.27 | Q15149 | Plectin | PLEC | apoptotic process, programmed cell death |
| 113 | 51.24 | 473 | 50.3 | 21.225 | 10 | 0.27 | P02794 | Ferritin heavy chain | FTH1 | cellular iron ion homeostasis |
| 114 | 11.346 | 480 | 13.5 | 28.518 | 5 | 0.25 | P08246 | Neutrophil elastase | ELANE | defense response, cytokine binding |
| 115 | 12.724 | 781 | 20.2 | 10.245 | 3 | 0.21 | P59666 | Neutrophil defensin 3 | DEFA3 | defense response |
| 116 | 247.9 | 1206 | 30.9 | 83.868 | 16 | 0.15 | P05164 | Myeloperoxidase | MPO | defense response, oxidation-reduction process |
| 117 | 323.31 | 3619 | 9.5 | 106.87 | 9 | 0.15 | B4DJ30 | Neutral alpha-glucosidase AB | GANAB | protein folding, post-translational protein modification |
| 118 | 95.465 | 772 | 24.3 | 76.625 | 15 | 0.13 | E7ER44 | Lactotransferrin | LTF | iron binding, regulation of cytokine production |

The table provides the protein names, numbers of identified peptides, scores, MS/MS counts, sequence coverages, molecular weights, calculated mean fold changes for iTRAQ and LF approaches, SwissProt accession numbers and the GO terms of the molecular function/biological process.
